# Arachidonic Acid Kills Staphylococcus aureus through a Lipid Peroxidation Mechanism

**DOI:** 10.1128/mBio.01333-19

**Published:** 2019-10-01

**Authors:** William N. Beavers, Andrew J. Monteith, Venkataraman Amarnath, Raymond L. Mernaugh, L. Jackson Roberts, Walter J. Chazin, Sean S. Davies, Eric P. Skaar

**Affiliations:** a Department of Pathology, Microbiology, and Immunology, Vanderbilt University Medical Center, Nashville, Tennessee, USA; b Department of Biochemistry, Vanderbilt University, Nashville, Tennessee, USA; c Department of Pharmacology, Vanderbilt University, Nashville, Tennessee, USA; d Department of Chemistry, Vanderbilt University, Nashville, Tennessee, USA; e Vanderbilt Institute for Infection, Immunology, and Inflammation, Vanderbilt University Medical Center, Nashville, Tennessee, USA; f Vanderbilt Institute of Chemical Biology, Vanderbilt University, Nashville, Tennessee, USA; University of Rochester

**Keywords:** isolevuglandins, arachidonic acid, MRSA, pathogenesis, polyunsaturated fatty acids, *Staphylococcus aureus*, wall teichoic acids

## Abstract

Staphylococcus aureus infects every niche of the human host. In response to microbial infection, vertebrates have an arsenal of antimicrobial compounds that inhibit bacterial growth or kill bacterial cells. One class of antimicrobial compounds consists of polyunsaturated fatty acids, which are highly abundant in eukaryotes and encountered by S. aureus at the host-pathogen interface. Arachidonic acid (AA) is one of the most abundant polyunsaturated fatty acids in vertebrates and is released in large amounts during the oxidative burst. Most of the released AA is converted to bioactive signaling molecules, but, independently of its role in inflammatory signaling, AA is toxic to S. aureus. Here, we report that AA kills S. aureus through a lipid peroxidation mechanism whereby AA is oxidized to reactive electrophiles that modify S. aureus macromolecules, eliciting toxicity. This process is rescued by cotreatment with antioxidants as well as in a S. aureus strain genetically inactivated for *lcpA* (USA300 *ΔlcpA* mutant) that produces lower levels of reactive oxygen species. However, resistance to AA stress in the USA300 *ΔlcpA* mutant comes at a cost, making the mutant more susceptible to β-lactam antibiotics and attenuated for pathogenesis in a murine infection model compared to the parental methicillin-resistant S. aureus (MRSA) strain, indicating that resistance to AA toxicity increases susceptibility to other stressors encountered during infection. This report defines the mechanism by which AA is toxic to S. aureus and identifies lipid peroxidation as a pathway that can be modulated for the development of future therapeutics to treat S. aureus infections.

## Abstract (precis)

Despite the ability of the human immune system to generate a plethora of molecules to control Staphylococcus aureus infections, S. aureus is among the pathogens with the greatest impact on human health. One class of host molecules toxic to S. aureus consists of polyunsaturated fatty acids. Here, we investigated the antibacterial properties of arachidonic acid, one of the most abundant polyunsaturated fatty acids in humans, and discovered that the mechanism of toxicity against S. aureus proceeds through lipid peroxidation. A better understanding of the molecular mechanisms by which the immune system kills S. aureus, and by which S. aureus avoids host killing, will enable the optimal design of therapeutics that complement the ability of the vertebrate immune response to eliminate S. aureus infections.

## INTRODUCTION

Staphylococcus aureus asymptomatically colonizes one in three humans, can infect every niche of the human host, and is the leading cause of Gram-positive sepsis, massively impacting human health (1, 2). Due to their close proximity and long history together, the human immune response generates a panoply of reactive molecules that are toxic to S. aureus and that limit the impact of bacterial infections (3–5). Expanding our understanding of the molecular mechanisms of toxicity by host molecules against S. aureus may further our understanding of why the immune system and current therapies fail to contain some S. aureus infections and enable the identification of new targets for antimicrobial development.

Polyunsaturated fatty acids (PUFA) represent one such host molecule with toxicity against S. aureus. These fatty acids are abundant in the membranes of host cells, but not bacterial cells, and are characterized by at least one bis-allylic carbon atom, making them highly susceptible to oxidation (6). PUFA are toxic not only to S. aureus *in vitro* (5) but also to Streptococcus pneumoniae (7), Acinetobacter baumannii (8), Cutibacterium acnes (9), Listeria monocytogenes (10), Pseudomonas aeruginosa (10), Neisseria gonorrhoeae (5), and Haemophilus influenzae (5), demonstrating the broad applicability of PUFA as antimicrobial compounds against both Gram-positive and Gram-negative pathogens. Mice fed a diet high in PUFA better survive S. aureus septicemia than mice fed a low-fat diet or a diet high in saturated fatty acids, indicating that PUFA are bactericidal against S. aureus *in vivo* (11). Despite the ability of PUFA to kill this diverse array of organisms, their mechanism of killing has not been defined, although studies investigating S. aureus resistance strategies have been performed. S. aureus utilizes efflux pumps to alleviate PUFA toxicity (12). Additionally, S. aureus can take up PUFA and esterify them into the phospholipid membrane, and the observed PUFA toxicity is dependent on the ability of S. aureus to esterify fatty acids into the membrane (13).

Arachidonic acid (AA) is a PUFA that is released during the inflammatory burst in large amounts by macrophages (14) and neutrophils (15). AA is metabolized enzymatically to potent signaling molecules, including prostaglandins (16), hydroxytetraenoic acids (17), and leukotrienes (18). Autoxidation of AA results in structurally similar but stereochemically diverse isoprostanes (19) in addition to various α/β-unsaturated carbonyls and free aldehydes (20–22). The latter molecules are electrophilic and react with nucleophilic groups of cellular macromolecules, including proteins, often resulting in deleterious effects on the target molecule. Several of these lipid peroxidation-derived molecules induce a stress response in S. aureus when added exogenously (23), but *in situ* generation and toxicity of these compounds in S. aureus have not yet been characterized.

The level of AA released and the amount of reactive oxygen species (ROS) generated at the host-pathogen interface led us to hypothesize that AA toxicity is mediated through lipid peroxidation. The ability of S. aureus to avoid AA toxicity was characterized by phenotypically selecting for a mutant resistant to AA killing (the USA300 *ΔlcpA* mutant strain). The role of LcpA in wall teichoic acid (WTA) biosynthesis led to the genetic and pharmacological characterization of the contribution of WTA to AA toxicity. While the USA300 *ΔlcpA* mutant exhibits *in vitro* resistance to AA, it is more sensitive to the β-lactam class of antibiotics and is not fully pathogenic in a murine model of infection. Here, we report that the mechanism mediating AA toxicity to S. aureus operates through lipid peroxidation, where AA is oxidized to compounds that kill S. aureus, and that this toxicity can be modulated by altering cellular ROS. This report identifies lipid peroxidation as a host pathway that can be perturbed as a part of future antimicrobial therapies against S. aureus.

## RESULTS

### AA kills S. aureus in a dose-dependent manner.

**FIG 1:**
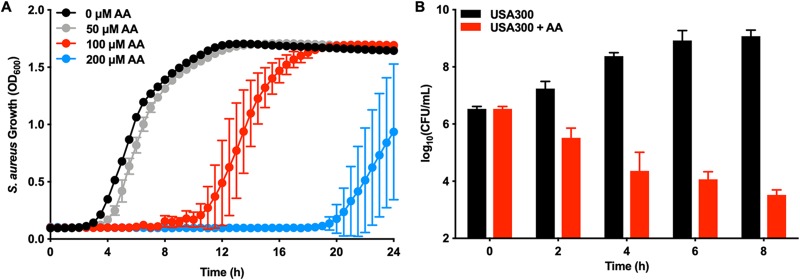
Arachidonic acid kills S. aureus in a dose-dependent manner. (A) USA300 was treated with various concentrations of AA, and growth was followed by monitoring optical density at 600 nm for 24 h. (B) USA300 was treated with 0 μM or 400 μM AA. At 0, 2, 4, 6, and 8 h post-AA treatment, S. aureus was serially diluted and plated on solid medium to enumerate viable bacteria. All data represent means ± standard deviations (SD) for measurements acquired in biological triplicate.

AA toxicity against S. aureus was determined by measuring staphylococcal growth in increasing concentrations of AA compared to an untreated control (Fig. 1A). AA is growth inhibitory to S. aureus, increasing the length of time that it takes the cells to reach a quantifiable density, in a dose-dependent manner. To determine whether AA is bactericidal or bacteriostatic, S. aureus was treated with and without AA and viability was assessed by plating for colony forming units (CFU) at various time points. Panel B of Fig. 1 shows that the CFU count measured for the AA-treated cells decreased over the course of the experiment, indicating that AA is bactericidal to S. aureus.

### Oxidative stress exacerbates AA toxicity in S. aureus.

**FIG 2:**
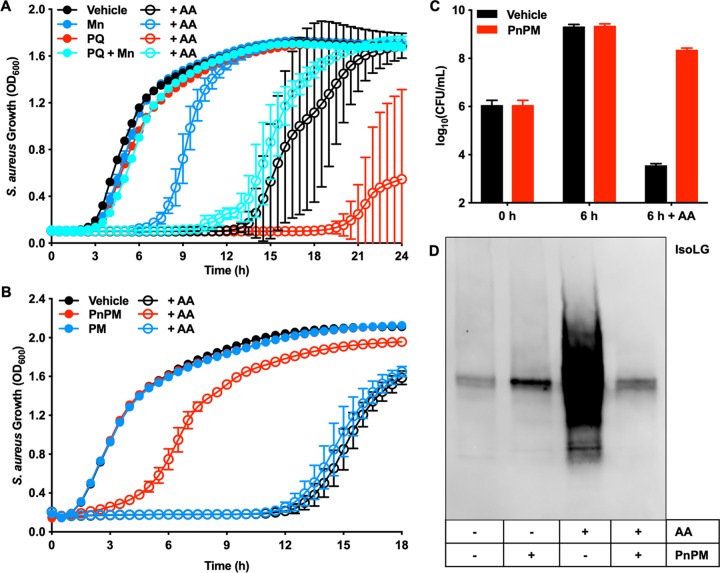
Arachidonic acid exerts its toxicity on S. aureus through a lipid peroxidation mechanism involving isolevuglandin formation. (A) USA300 was treated with 0 μM or 100 μM AA in combination with 0 μM or 250 μM Mn, which S. aureus uses as an antioxidant, and 0 μM or 250 μM paraquat (PQ), which generates reactive oxygen species, and growth was followed by monitoring optical density at 600 nm for 24 h. (B) USA300 was treated with 0 μM or 200 μM AA in combination with 0 mM or 1 mM 5′-*O*-pentyl-pyridoxamine (PnPM), a membrane-permeative lipid dicarbonyl scavenger, and 0 μM or 1 mM pyridoxamine (PM), a membrane-impermeative lipid dicarbonyl scavenger, and growth was followed by monitoring optical density at 600 nm for 18 h. (C) USA300 was treated with 0 μM or 100 μM AA and 0 mM or 1 mM PnPM for 6 h, and then the samples were serially diluted and plated on solid medium to enumerate viable bacteria. (D) USA300 was treated with 0 μM or 400 μM AA and 0 mM or 5 mM PnPM for 6 h, and the protein lysates were isolated, separated by SDS-PAGE, and probed with an antibody that recognizes IsoLG protein after translational modifications. All data represent means ± SD for measurements acquired in biological triplicate.

To test the hypothesis that the mechanism of AA toxicity is driven by oxidative stress, S. aureus was treated with AA and with nontoxic levels of the ROS-generating agent paraquat. Nontoxic concentrations of paraquat increased the amount of lipid peroxidation in S. aureus without contributing directly to the observed toxicity, allowing us to distinguish killing by ROS from killing by AA lipid peroxidation products. These experiments were also performed in the presence or absence of the antioxidant manganese (Mn) (24). Panel A of Fig. 2 shows that Mn reduced AA toxicity whereas paraquat increased AA toxicity. Combining paraquat, Mn, and AA treatments resulted in a growth phenotype similar to that seen with bacteria treated with AA alone, indicating that the ROS generated by paraquat was quenched by Mn. We also tested the antioxidant α-tocopherol (vitamin E), which is a radical-chain-terminating antioxidant, halting lipid peroxidation (25). Figure S1 shows that α-tocopherol cotreatment with AA eliminated AA toxicity. Together, these data support the hypotheses that AA toxicity is modulated by cellular ROS levels and that lipid peroxidation products represent the species toxic to S. aureus.

10.1128/mBio.01333-19.1 FIG S1 A lipid peroxidation-terminating antioxidant alleviates AA toxicity in S. aureus. USA300 was treated with various concentrations of AA and either 0 μM or 80 μM α-tocopherol, a radical-chain-terminating antioxidant, and growth was followed by monitoring optical density at 600 nm for 24 h. Data represent means ± standard deviations (SD) for measurements acquired in biological triplicate. Download FIG S1, TIF file, 14.5 MB. Copyright © 2019 Beavers et al. 2019 Beavers et al. This content is distributed under the terms of the Creative Commons Attribution 4.0 International license.

### Isolevuglandins are AA metabolites that kill S. aureus.

During lipid peroxidation, AA is oxidized to many electrophilic species, including isolevuglandins (IsoLG), which have well-defined toxicity in eukaryotic cells (22, 26). Thus, tools are available to measure IsoLG-dependent posttranslational modifications (PTM) (27) and to specifically scavenge IsoLG and related lipid dicarbonyls, alleviating their toxicity (28). S. aureus was treated with AA in combination with either of two lipid dicarbonyl scavengers, pyridoxamine (PM) or 5′-*O*-pentyl-pyridoxamine (PnPM), both of which are 3 orders of magnitude more reactive with IsoLG than the ε-amine of lysine and prevent the formation of toxic PTM (29) (see Fig. S2 in the supplemental material). PM is polar and does not cross lipid membranes, while PnPM is hydrophobic and readily diffuses across membranes, allowing us to both determine if IsoLG are involved in AA toxicity against S. aureus and define whether AA oxidation occurs intracellularly or extracellularly. Figure 2B shows that PnPM rescued AA toxicity whereas PM did not, indicating that reactive lipid dicarbonyls such as IsoLG mediate the toxic effects of AA and that AA oxidation occurs intracellularly. We also measured the ability of PnPM to rescue AA-mediated killing of S. aureus and found that cotreatment with AA and PnPM resulted in a nearly 5-log increase in CFU after 6 h of growth compared to the cells treated with AA alone, further indicating that IsoLG and related dicarbonyls are AA-derived species that are toxic to S. aureus (Fig. 2C). Complete rescue of AA toxicity was not observed in any of these scavenging experiments, presumably because AA is oxidized to many reactive molecules (such as 4-hydroxy-2-nonenal) that also have cellular toxicity but that do not react with the scavengers tested here.

10.1128/mBio.01333-19.2 FIG S2 Schematic of lipid dicarbonyl scavenger activity. (A and B) Lipid dicarbonyl scavengers that cannot diffuse across the membrane, i.e., pyridoxamine (PM) (A), and that can diffuse across the membrane, i.e., 5′-*O*-pentyl-pyridoxamine (PnPM) (B), were used to determine whether IsoLG represent the species responsible for AA toxicity to S. aureus and also where the IsoLG are generated, inside or outside the S. aureus cell. (C) Both PM and PnPM have a primary amine that is 1,000-fold more reactive with IsoLG than the ε-amine of lysine. Therefore, the scavengers preferentially form a nontoxic adduct, preventing the formation of toxic IsoLG adducts and alleviating AA toxicity against S. aureus. Download FIG S2, TIF file, 14.5 MB. Copyright © 2019 Beavers et al. 2019 Beavers et al. This content is distributed under the terms of the Creative Commons Attribution 4.0 International license.

To confirm the role of IsoLG in S. aureus AA toxicity, an antibody (Ab) against the IsoLG PTM (27) was used to probe S. aureus lysates treated with and without AA and PnPM. Figure 2D shows that the IsoLG PTM level were increased in S. aureus treated with AA and were reduced to baseline levels when PnPM was coadministered with AA. In total, these data suggest that AA is oxidized to IsoLG within S. aureus, adducting cellular proteins and eliciting its toxicity, and that this toxicity can be modulated by scavenging IsoLG.

### LcpA sensitizes S. aureus to AA toxicity.

**FIG 3:**
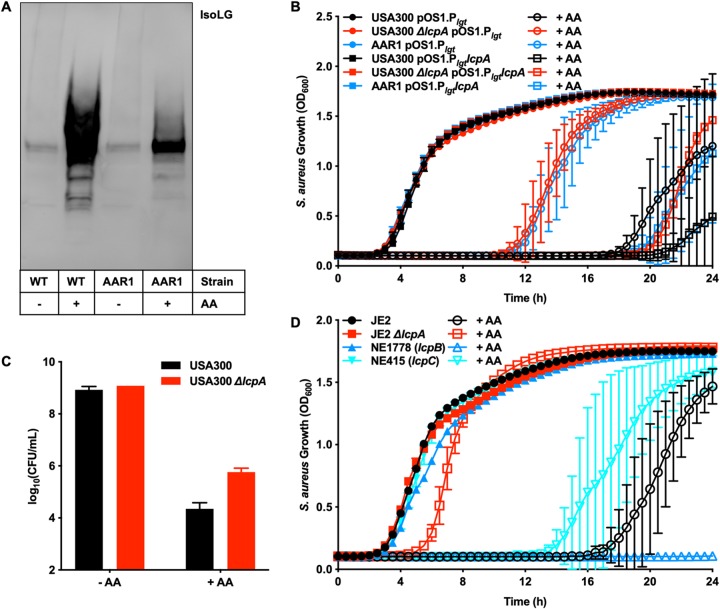
Deletion of *lcpA* protects S. aureus from IsoLG-mediated killing by AA. (A) Wild-type strain USA300 or AAR1 was treated with 0 μM or 400 μM AA, and the protein lysates were isolated, separated by SDS-PAGE, and probed with an antibody that recognizes IsoLG protein after translational modifications. (B) In addition to AAR1, which produces an inactive LcpA, the USA300 *ΔlcpA* strain, which has the entire gene deleted, was created. USA300, AAR1, and USA300 *ΔlcpA* strains with (pOS1.P*_lgt_lcpA*) and without (pOS1.P*_lgt_*) overexpression of *lcpA* were treated with 0 μM or 100 μM AA, and growth was followed by monitoring optical density at 600 nm for 24 h. (C) Strains USA300 and USA300 *ΔlcpA* were treated with 0 μM or 100 μM AA for 6 h, and then the samples were serially diluted and plated on solid medium to enumerate viable bacteria. (D) Each *lcp* gene was individually deleted or inactivated, and the resulting strains were tested for resistance to AA toxicity. Each strain was combined with 0 μM or 100 μM AA, and growth was followed by monitoring optical density at 600 nm for 24 h. All data represent means ± SD for measurements acquired in biological triplicate.

Selection for bacterial mutants resistant to a stressor represents a powerful tool used to understand the bacterial processes that contribute to the toxicity of the compound of interest. AA-resistant strains were selected for by passaging of USA300 on solid medium containing AA and characterizing the colonies that survived. The mutational frequency calculated during these experiments was 0.0000089. AA-resistant mutant 1 (AAR1), which has the mutation K253* in the protein named LytR-CpsA-Psr A (LcpA), was selected for further characterization. The LcpA K253* mutation is the only nonsynonymous mutation in this strain and results in a quarter of the catalytic domain being deleted. LcpA takes WTA from the membrane where they are synthesized and ligates them into the cell wall (30). Figure 3A shows that AAR1 had less IsoLG PTM than USA300 under conditions of treatment with AA, indicating that the inactivation of LcpA reduced the levels of lipid peroxidation and thus of IsoLG protein modification. Therefore, we hypothesized that LcpA sensitizes S. aureus to AA toxicity.

To test if the AAR1 mutation results in an inactive LcpA, *lcpA* was deleted and the resulting strain, the USA300 *ΔlcpA* mutant, was tested for AA resistance. Figure 3B shows that strain AAR1 and the USA300 *ΔlcpA* mutant had the same growth phenotype and were resistant to AA toxicity. Additionally, the toxicity phenotype was complemented in both AAR1 and the USA300 *ΔlcpA* mutant by providing *lcpA* in *trans* (Fig. 3B). Finally, the USA300 *ΔlcpA* mutant showed higher levels of viable bacteria than strain USA300 under conditions of treatment with AA (Fig. 3C). S. aureus encodes three Lcp enzymes, LcpA, LcpB, and LcpC, which appear to have some redundant functions based on glycan staining of the S. aureus cell (31). Although *lcpA* and *lcpB* knockout strains have similar WTA phenotypes (31), only the deletion of *lcpA* confers resistance to AA toxicity. Inactivation of *lcpB* exacerbates AA toxicity (32), while the growth phenotype resulting from inactivation of *lcpC* was similar to that seen with strain JE2, the parental USA300 strain cured for erythromycin resistance (Fig. 3D). These data show that *lcpA* sensitized S. aureus to AA toxicity, that inactivating *lcpA* resulted in a strain more resistant to AA killing, and that the AA resistance was LcpA specific and not conserved across all Lcp enzymes.

### Inactivating *lcpA* increases MRSA β-lactam antibiotic sensitivity.

**FIG 4:**
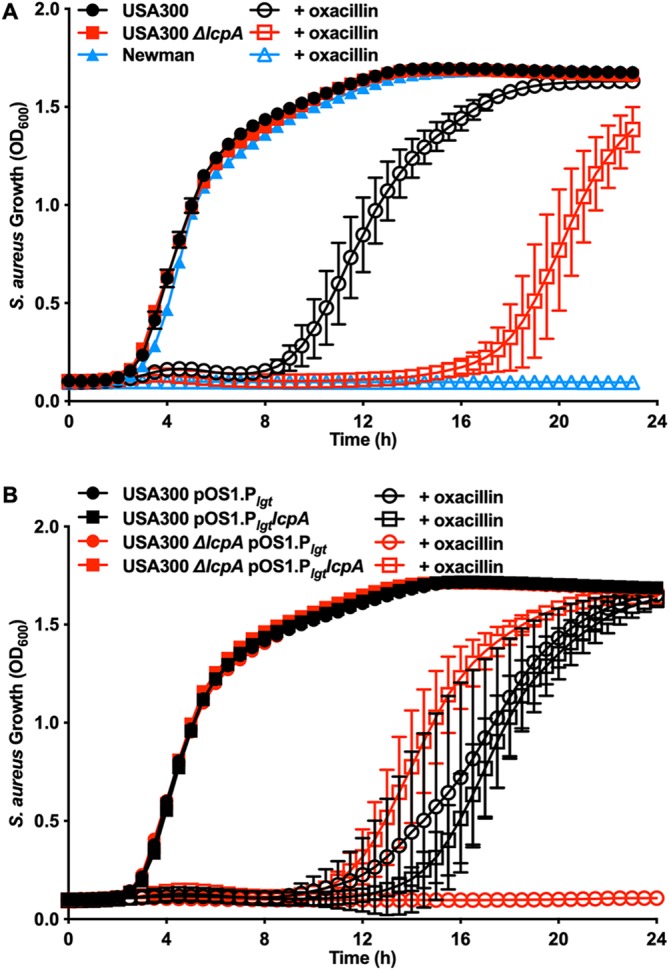
Deletion of *lcpA* enhances susceptibility of MRSA to β-lactam antibiotics. (A) USA300 and the USA300 *ΔlcpA* mutant, both MRSA strains, and Newman, an MSSA strain, were treated with 0 or 2 μg/ml oxacillin, and growth was followed by monitoring optical density at 600 nm for 24 h. Data represent means ± SD for measurements acquired in biological quadruplicate. (B) Strains USA300 and USA300 *ΔlcpA* with and without the constitutive expression of *lcpA* from the pOS1 vector were treated with 0 or 4 μg/ml oxacillin, and growth was followed by monitoring optical density at 600 nm for 24 h. Data represent means ± SD for measurements acquired in biological triplicate.

Altering the cell wall in methicillin-resistant S. aureus (MRSA) affects its ability to protect against the antimicrobial activity of β-lactam antibiotics. Therefore, we tested the hypothesis that deletion of *lcpA* would convert an MRSA strain into a methicillin-sensitive S. aureus (MSSA) strain. USA300, an MRSA strain, was more resistant to growth inhibition by oxacillin than Newman, an MSSA strain (Fig. 4A). Inactivating *lcpA* increased USA300 sensitivity to oxacillin but did not convert the MRSA strain into an MSSA strain (Fig. 4A). Consistent with previous work (30), β-lactam sensitivity was able to be complemented by providing *lcpA in trans*, where the strains treated with oxacillin that express *lcpA* had lower sensitivity to oxacillin than the USA300 *ΔlcpA* mutant (Fig. 4B). These data indicate that whereas *lcpA* inactivation protected against AA-mediated killing, this mutation caused cells to become more sensitive to β-lactam antibiotics.

### LcpA does not alter the S. aureus barrier to exclude AA.

**FIG 5:**
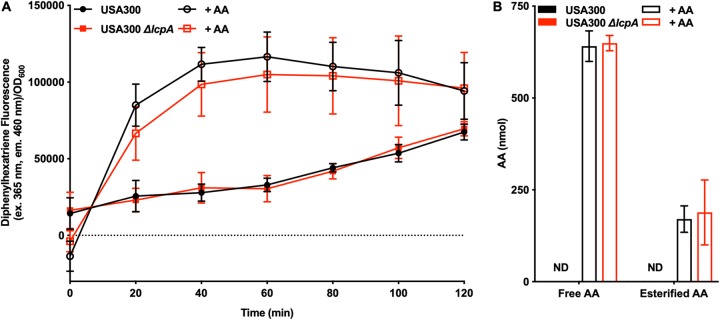
Deleting *lcpA* does not alter the S. aureus barrier function. (A) Strains USA300 and USA300 *ΔlcpA* were combined with 0 μM or 100 μM AA and 0 μM or 100 μM diphenylhexatriene, a molecule that fluoresces in the cell membrane. Membrane permeability was quantified over 2 h by monitoring the fluorescence of diphenylhexatriene (excitation [ex.], 365 nm; emission [em.], 460 nm). (B) Strains USA300 and USA300 *ΔlcpA* were treated with 0 μM or 400 μM AA for 1 h. Following AA treatment, the membranes were extracted. Free AA was quantified in each sample, and then, following base hydrolysis, total AA was quantified for each sample. Esterified AA levels were calculated by subtracting the amount of free AA from the total amount of AA in each sample (ND = not detected). All data represent means ± SD for measurements acquired in biological quadruplicate.

The membrane permeability of S. aureus was tested using 1,6-diphenyl-1,3,5-hexatriene (DPH), a compound that fluoresces only in the lipid membrane (33). As shown in Fig. 5A, DPH was incorporated into the membrane equally in USA300 and the USA300 *ΔlcpA* mutant, indicating that the presence of LcpA did not change the permeability of the cell wall or the permeability of the membrane. Interestingly, the strains treated with AA were found to have a higher level of membrane permeability than the untreated strains (Fig. 5A), which is consistent with the observation that the *cis* double bonds of unsaturated fatty acids, such as AA, increase membrane fluidity (34).

In addition to testing membrane permeability, we quantified the amount of AA in each strain. No AA was detected in the strains that were not treated with AA (Fig. 5B), indicating that the lack of AA in S. aureus may be responsible for its increased sensitivity to killing by AA. Following treatment with AA, the amounts of AA quantified both as the free fatty acid and as the fatty acid esterified into the phospholipid bilayer did not differ between the USA300 strain and the USA300 *ΔlcpA* mutant (Fig. 5B). In total, these experiments demonstrate that the deletion of *lcpA* did not alter the S. aureus barrier with respect to AA uptake.

### Wall teichoic acids protect S. aureus from toxicity by AA.

**FIG 6:**
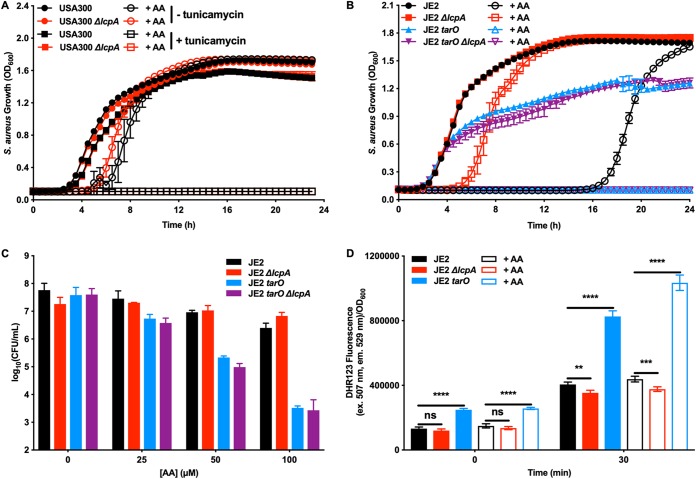
Inactivating wall teichoic acid biosynthesis enhances S. aureus susceptibility to AA toxicity. (A) Strains USA300 and USA300 *ΔlcpA* were treated with 0 μM or 50 μM AA and 0 μg/ml or 2 μg/ml tunicamycin, an inhibitor of WTA biosynthesis, and growth was followed by monitoring optical density at 600 nm for 24 h. (B) Strains JE2, JE2 *ΔlcpA*, JE2 *tarO*, and JE2 *ΔlcpA tarO* were treated with 0 μM or 100 μM AA, and growth was followed by monitoring optical density at 600 nm for 24 h. (C) Strains JE2, JE2 *ΔlcpA*, JE2 *tarO*, and JE2 *ΔlcpA tarO* were treated with various concentrations of AA for 3 h, and then the samples were serially diluted and plated on solid medium to enumerate viable bacteria. All data represent means ± SD for measurements acquired in biological triplicate. (D) Strains JE2, JE2 *ΔlcpA*, and JE2 *tarO* were combined with 0 μM or 100 μM AA and 0 or 50 μM dihydrorhodamine 123, a molecule that fluoresces after reacting with ROS. ROS generation was quantified over 30 min by monitoring the fluorescence of dihydrorhodamine 123 (ex., 507 nm; em., 529 nm). Data represent means ± SD for measurements acquired in biological triplicate on two separate days combined (*n* = 6), and statistical significance was determined by one-way analysis of variance (ANOVA; **, *P* < 0.01; ***, *P* < 0.001; ****, *P* < 0.0001).

LcpA ligates WTA into the cell wall, and we identified another mutation that leads to resistance to AA toxicity in TarJ F287L; therefore, we tested the role of WTA in S. aureus resistance to AA toxicity. Tunicamycin inhibits TarO, the first enzyme in the WTA biosynthetic pathway (35), sensitizing S. aureus to β-lactam antibiotics at 0.02 μg/ml tunicamycin and inhibiting S. aureus growth at 10 μg/ml tunicamycin (35). At a concentration below the growth inhibitory concentration but above the sensitizing concentration (2 μg/ml), tunicamycin combined with AA completely inhibited S. aureus growth independently of *lcpA* (Fig. 6A), suggesting that WTA synthesis was necessary to prevent AA killing but that the ligation of WTA into the cell wall by LcpA sensitized S. aureus to AA killing.

To confirm the role of WTA in S. aureus AA toxicity, we used a strain that produces an inactive TarO, strain JE2 *tarO^30^*. In concordance with the tunicamycin data presented in Fig. 6A, JE2 *tarO* did not grow under conditions of treatment with AA, independently of *lcpA* (Fig. 6B). AA killing of JE2 *tarO* also proceeded through a lipid peroxidation mechanism, as an increase in IsoLG was observed upon treatment with AA (Fig. S3A) and cotreatment of α-tocopherol with AA was protective to the cells (Fig. S3B). As shown in Fig. 6C, concentrations of AA that minimally reduced CFU level in JE2 or in the JE2 *ΔlcpA* mutant reduced the size of viable colonies in JE2 *tarO* by multiple orders of magnitude independently of *lcpA*. In total, these data show that WTA are necessary to protect S. aureus from AA toxicity and that an inactive LcpA protects S. aureus from AA killing only when WTA biosynthesis is fully functioning.

10.1128/mBio.01333-19.3 FIG S3 AA kills JE2 *tarO* through a lipid peroxidation mechanism. (A) The JE2, JE2 Δ*lcpA*, and JE2 *tarO* strains were treated with 0 μM or 400 μM AA for 6 h, and the protein lysates were isolated, separated by SDS-PAGE, and probed with an antibody that recognizes IsoLG protein after translational modifications. (B) JE2 *tarO* was treated with 0 μM or 25 μM AA and 0 μM or 80 μM α-tocopherol, a radical-chain-terminating antioxidant, and growth was followed by monitoring optical density at 600 nm for 24 h. Data represent means ± SD for measurements acquired in biological triplicate. Download FIG S3, TIF file, 14.5 MB. Copyright © 2019 Beavers et al. 2019 Beavers et al. This content is distributed under the terms of the Creative Commons Attribution 4.0 International license.

### Cellular ROS generation determines S. aureus susceptibility to AA toxicity.

Since the mechanism by which AA kills S. aureus proceeds through lipid peroxidation, the hypothesis that cellular ROS contributes to AA toxicity was tested by measuring cellular ROS in JE2, the JE2 *ΔlcpA* mutant, and JE2 *tarO*, with and without AA treatment (Fig. 6D). JE2 *tarO* produced more ROS both with and without AA treatment than either of the other strains, which is consistent with this strain being more susceptible to killing by AA. The JE2 *ΔlcpA* mutant produced less ROS than strain JE2, correlating with this strain having lower levels of IsoLG PTM and being more resistant to AA killing. These data confirm that cellular ROS is a determinant of AA toxicity in S. aureus, supporting the model whereby the generation of lipid peroxidation species represents the mechanism of killing by AA.

### Deletion of *lcpA* reduces neutrophil killing but slows growth following coculture with neutrophils.

**FIG 7:**
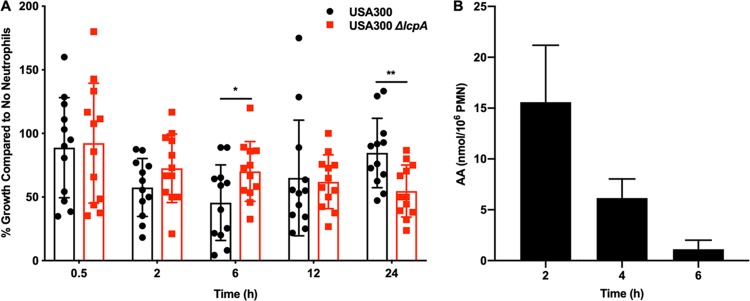
Strains USA300 and USA300 *ΔlcpA* are differentially killed by murine neutrophils. (A) Killing by neutrophils isolated from 7-week-old female BALB/cJ mice was assessed by comparisons between strains USA300 and USA300 *ΔlcpA*. Bacteria were combined with neutrophils (MOI = 2) for 0.5, 2, 6, 12, or 24 h. Following incubation, the samples were serially diluted and plated on solid medium to enumerate viable bacteria. Data represent means ± SD of combined results from neutrophils isolated from four mice, and neutrophil killing of each isolate was determined in bacterial biological triplicate (*n* = 12). Statistical significance was determined by *t* test (*, *P* < 0.05; **, *P* < 0.01). (B) Neutrophils isolated from 8-week-old female BALB/cJ mice were activated by heat-killed USA300 (MOI = 1) for 2, 4, or 6 h. The AA released from the phospholipid bilayer during activation was quantified at each time point. Data represent means ± SD of results from neutrophils isolated from four mice.

Neutrophils are central to the innate immune response during S. aureus infections, and AA is released from the membranes of neutrophils during the oxidative burst. Therefore, the hypothesis that the USA300 *ΔlcpA* mutant is more resistant to neutrophil killing than USA300 was tested. After a 6-h coculture with neutrophils, the USA300 *ΔlcpA* mutant showed increased bacterial growth compared to USA300 (Fig. 7A). The difference at the 6-h time point is consistent with many components of the oxidative burst being fully deployed, including AA release from the phospholipid membrane, HOCl generation as a part of the oxidative burst, and calprotectin release as a part of nutritional immunity (36). However, by 24 h, after the neutrophils have died, the phenotype reversed, with the USA300 *ΔlcpA* mutant lagging USA300 in bacterial growth, suggesting that LcpA contributes to bacterial growth following neutrophil-induced stress (Fig. 7A). As shown in Fig. 7B, neutrophils activated by heat-killed S. aureus released AA at levels high enough to be bactericidal against S. aureus at 2, 4, and 6 h after activation, consistent with the rescue seen at 6 h in the USA300 *ΔlcpA* mutant. No differences in HOCl killing (Fig. S4A) or calprotectin growth inhibition (Fig. S4B) were observed between the strains, leading to the conclusion that the increased killing of the USA300 *ΔlcpA* mutant at 6 h might have resulted from AA toxicity. The USA300 *ΔlcpA* mutant induced an oxidative burst from neutrophils similar to that induced by USA300 (Fig. S5A and B), indicating that differences in neutrophil activation do not explain the observed killing phenotypes. These data demonstrate that deletion of *lcpA* contributes to the ability of S. aureus to survive killing by neutrophils at times consistent with the release of AA from the membrane but that the deletion of *lcpA* comes at a fitness cost to S. aureus after neutrophil-induced stress.

10.1128/mBio.01333-19.4 FIG S4 The USA300 and USA300 *ΔlcpA* strains are not differentially susceptible to neutrophil-derived antimicrobial compounds. (A) The USA300 and USA300 *ΔlcpA* strains were treated with various concentrations of HOCl for 30 min, and then the samples were serially diluted and plated on solid medium to enumerate viable bacteria. Data represent means ± SD for measurements acquired in biological triplicate. (B) The USA300 and USA300 *ΔlcpA* strains were treated with 0 μg/ml or 800 μg/ml calprotectin, a nutrient metal chelator, and growth was followed by monitoring optical density at 600 nm for 24 h. Data represent means ± SD for measurements acquired in biological quadruplicate. Download FIG S4, TIF file, 14.5 MB. Copyright © 2019 Beavers et al. 2019 Beavers et al. This content is distributed under the terms of the Creative Commons Attribution 4.0 International license.

10.1128/mBio.01333-19.5 FIG S5 The USA300 and USA300 *ΔlcpA* strains do not differentially activate murine neutrophils. (A) Neutrophil activation was measured by flow cytometry for each strain using dihydrorhodamine 123 fluorescence and neutrophils isolated from 7-week-old female BALB/cJ mice. Bacteria were combined with neutrophils (MOI = 10) for 30 or 60 min. Data represent means ± SD of combined results from neutrophils isolated from six mice. Statistical significance was determined by *t* test. (B) Flow gating scheme describing the quantification of neutrophil ROS generated in response to coculture of neutrophils and either USA300 or the USA300 *ΔlcpA* mutant at an MOI of 10. Download FIG S5, TIF file, 14.5 MB. Copyright © 2019 Beavers et al. 2019 Beavers et al. This content is distributed under the terms of the Creative Commons Attribution 4.0 International license.

### LcpA is required for full S. aureus colonization of the murine brain, kidneys, and spleen.

**FIG 8:**
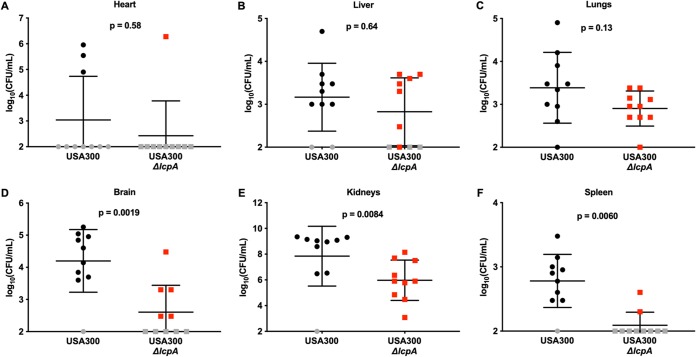
S. aureus lacking *lcpA* is attenuated for pathogenesis compared to USA300 in a murine model of systemic infection. Female 7-week-old BALB/cJ mice were infected retro-orbitally with USA300 or the USA300 *ΔlcpA* mutant. After 4 days of monitoring, the (A) heart, (B) liver, (C) lungs, (D) brain, (E) kidneys, and (F) spleen were harvested, homogenized, serially diluted, and plated on solid medium to enumerate viable bacteria. Gray symbols indicate that bacterial burdens were below the limit of detection; for those bacterial burdens, the values corresponding to the limit of detection were used for statistical analyses. Data represent means ± SD (*n* = 10 mice infected per bacterial strain), and *P* values were calculated by Mann-Whitney test.

Seven-week-old female BALB/cJ mice were infected systemically with USA300 or the USA300 *ΔlcpA* mutant and monitored for 4 days, and then the organs were harvested for bacterial CFU enumeration. The mice infected with USA300 lost more weight than the mice infected with the USA300 *ΔlcpA* mutant, indicating that the USA300-infected mice were sicker and had a more difficult time clearing the infection (Fig. S6). No bacterial burden differences between the strains were observed in the heart (Fig. 8A), liver (Fig. 8B), or lungs (Fig. 8C). However, the USA300 *ΔlcpA* mutant was less virulent than the parental USA300 strain in the brain (Fig. 8D), kidneys (Fig. 8E), and spleen (Fig. 8F). These data demonstrate that *lcpA* is necessary for full virulence, consistent with the USA300 *ΔlcpA* mutant having reduced growth following neutrophil stress. In total, these data indicate that mutations in cell wall machinery can confer *in vitro* and short-term resistance against the host stressors that S. aureus encounters during infection but that the complete cell wall assembly machinery is necessary for full S. aureus virulence.

10.1128/mBio.01333-19.6 FIG S6 Mice infected with USA300 lose more weight than mice infected with the USA300 *ΔlcpA* mutant. Mice infected retro-orbitally with USA300 or the USA300 *ΔlcpA* mutant were monitored and weighed daily. Weight loss was used as an indicator of how healthy the mice were following infection. Data represent means ± SD (*n* = 10 mice infected per bacterial strain), and *P* values were calculated by Mann-Whitney test. Download FIG S6, TIF file, 14.5 MB. Copyright © 2019 Beavers et al. 2019 Beavers et al. This content is distributed under the terms of the Creative Commons Attribution 4.0 International license.

## DISCUSSION

PUFA biosynthetic enzymes have not been annotated in S. aureus, and we have not detected PUFA at biologically relevant levels in S. aureus, suggesting that S. aureus encounters PUFA only at the host-pathogen interface. AA is a PUFA that is released in large amounts during the oxidative burst. AA has three bis-allylic carbon atoms, making it particularly susceptible to oxidation to various bioactive lipid peroxidation products, including prostaglandins, isoprostanes, leukotrienes, and hydroxytetraenoic acids (6). Autoxidation of AA by cellular ROS also results in myriad reactive α,β-unsaturated carbonyls and free aldehydes, including IsoLG, that modify nucleophilic groups of macromolecules, eliciting deleterious effects to cellular machinery (20–22).

Here, we report that AA treatment of S. aureus kills the pathogen in a ROS-dependent manner. Nontoxic levels of ROS exacerbate the toxicity of AA, indicating that the concentrations of AA encountered by S. aureus at the host-pathogen interface, combined with the large amounts of oxidants generated during the host oxidative burst, can cooperate to kill S. aureus. Further confirming the role of lipid peroxidation in AA toxicity, α-tocopherol, a radical-chain-terminating antioxidant, prevents the killing of S. aureus by AA. This is consistent with a previous study demonstrating that toxicity by AA is abrogated in S. aureus by the administration of ascorbate, an antioxidant (5). The oxidation of AA to IsoLG (and potentially other lipid dicarbonyls) results in the macromolecular damage responsible for the observed AA toxicity. Unlike many other electrophilic modifications, IsoLG adducts are irreversible and extremely hydrophobic, making them particularly potent PTM for altering cellular function (21, 22). Toxicity and generation of IsoLG have been extensively explored in eukaryotic cells (37, 38) but are not well understood in bacteria. The use of hydrophobic dicarbonyl scavengers allowed us to determine that the oxidation of AA occurs after AA is taken into S. aureus and not outside the cell. This is a particularly important distinction because the level of ROS generated during normal bacterial cellular processes is much lower than that generated during the oxidative burst. Therefore, the bactericidal role of lipid peroxidation products is much greater at the host-pathogen interface.

S. aureus strains spontaneously resistant to AA killing were selected for *in vitro*, and the inactivation of *lcpA* conferred S. aureus resistance to AA toxicity by reducing the amount of IsoLG PTM. Though S. aureus has three Lcp enzymes with redundant WTA functions (31), only the deletion of *lcpA* resulted in a strain resistant to AA toxicity. The *lcpB*-deleted strain, which had the WTA phenotype most similar to that of the *lcpA*-deleted strain (31), exhibited the entirely opposite AA toxicity phenotype, where this strain was found to be extremely sensitive to AA stress. These data indicate more-diverse roles beyond insertion of WTA into the cell wall for the Lcp class of enzymes in S. aureus host defense. Deleting *lcpA* reduces WTA levels but does not eliminate WTA due to the presence of multiple *lcp* genes (31), and so the hypothesis that deletion of *lcpA* alters the S. aureus barrier and changes the susceptibility to toxic molecules was tested. In experiments that evaluated envelope permeability and quantified total AA levels, no differences were observed, indicating that a change in the S. aureus barrier was not responsible for the differential killing phenotypes observed.

WTA biosynthesis was modulated by chemically and genetically inhibiting the WTA biosynthetic machinery. This resulted in increased sensitivity to AA toxicity, which is consistent with the finding that linoleic acid, another abundant host PUFA, is more toxic to S. aureus when *tarO* is inactivated (39). A previous study showed that *tarO*-deficient S. aureus is more susceptible to detergent killing than the wild-type (WT) strain (40). The concentration of AA needed to kill JE2 *tarO* was 50-fold lower than the concentration of detergent used in the previous study, and the killing of JE2 *tarO* was rescued with α-tocopherol, indicating that the killing observed in our study occurred through lipid peroxidation. These data also highlight the complicated role for WTA in the modulation of AA toxicity. AA toxicity is enhanced in strains treated with tunicamycin as well as in JE2 *tarO*, and both strains treated with tunicamycin and JE2 *tarO* are deficient in WTA biosynthesis. The status of LcpA, the enzyme that ligates WTA into the cell wall, does not affect AA sensitivity in WTA-null strains. However, a functional copy of LcpA makes S. aureus more susceptible to AA killing in strains that synthesize WTA. These data show that PUFA treatment in conjunction with administration of current antimicrobials that inhibit WTA biosynthesis, such as tunicamycin, is a potential strategy for treating S. aureus infections.

Cellular ROS levels directly modulate the ability of AA to kill S. aureus, so we hypothesized that the increased toxicity observed in JE2 *tarO* was due to an increase in intracellular ROS, resulting in increased lipid peroxidation. Using a ROS-reactive dye, we determined that JE2 *tarO* had increased levels of ROS compared to both JE2 and the JE2 *ΔlcpA* mutant. These data are consistent with the finding that the strain inactivated for *tarO* was the most susceptible to killing by AA. In concordance, the JE2 *ΔlcpA* mutant showed deceased levels of ROS compared to JE2, and was more resistant to AA killing. As mentioned above, small, nontoxic perturbations of cellular ROS levels have drastic effects on AA toxicity. These data indicate that the JE2 *ΔlcpA* mutant reduces the cellular ROS levels by an unknown mechanism, reducing the levels of lipid peroxidation and the resulting AA toxicity for those cells.

To determine if the *in vitro* phenotypes showing that the USA300 *ΔlcpA* mutant was resistant to AA toxicity led to increased virulence *in vivo*, we tested the role of LcpA in neutrophil killing and pathogenesis. The USA300 *ΔlcpA* mutant was more resistant than USA300 to neutrophil killing at 6 h postinoculation. Since there was no difference between USA300 and the USA300 *ΔlcpA* mutant in the levels of susceptibility to killing by HOCl and growth inhibition by calprotectin, representing two major components of the neutrophil used to control bacterial infection, the phenotype is potentially attributed to AA killing at this time point. At 24 h, the recovery of USA300 from neutrophil coculture was better than that seen with the USA300 *ΔlcpA* mutant, which is consistent with the *in vivo* murine infection data showing that the USA300 *ΔlcpA* mutant was less virulent in the brain, kidneys, and spleen at 4 days postinfection. Taking the data together, the deletion of *lcpA* conferred resistance to a host molecule that is an important part of the immune response but the totality of the immune response eventually overcame this resistance, such that this mutant was more susceptible to host killing or less able to overcome cellular damage by the host.

In this study, the oxidation of AA into reactive electrophiles was identified as the mechanism by which AA kills S. aureus and a mutation in *lcpA* that makes S. aureus resistant to AA killing was discovered. However, this mutant is more susceptible to antibiotic killing and less pathogenic *in vivo*. We expect that mutations in WTA biosynthetic machinery are not likely to arise *in vivo* because they make S. aureus less pathogenic. This, combined with the fact that AA has very low toxicity in humans, makes AA or other species that undergo lipid peroxidation potentially useful for treating S. aureus infections both alone and in combination with other treatments, such as those employing inhibitors of WTA biosynthesis. Initially, the most straightforward S. aureus infection niche to target is the skin, since the bacteria are accessible to topical balms and because delivery of AA to internal sites of infection is difficult due to the host mechanisms of AA uptake and usage. The best ways to exploit lipid peroxidation as a systemic treatment are to identify the targets of lipid electrophiles and define the role that those targets play in S. aureus pathogenesis. Detailed knowledge of the key lipid electrophile targets will allow even more options that can be used to address therapeutic weaknesses and be exploited to make AA released at the host-pathogen interface more toxic to S. aureus. In fact, a recent study used blue light treatment of S. aureus to produce cellular ROS, increasing levels of lipid peroxidation products and killing S. aureus (41). The combined results of the studies show that modulation of lipid peroxidation at the host-pathogen interface is an attractive and exciting new area to investigate for novel therapeutic strategies to combat S. aureus infections.

## MATERIALS AND METHODS

### Materials.

Bacterial strains (Table 1), plasmids (Table 2), and primers (Table 3) are listed in their respective tables. All laboratory plasticware was obtained from USA Scientific, Ocala, FL, or Corning, Corning, NY. All S. aureus growth experiments were performed on tryptic soy agar (TSA) or in tryptic soy broth (TSB) (Becton Dickinson, Franklin Lakes, NJ). All fatty acids were obtained from Cayman Chemical, Ann Arbor, MI, and diluted to 1,000× stocks in ethanol. All other chemicals were obtained from Sigma St. Louis, MO, unless otherwise noted. All PCR procedures were performed using 2× Phusion HF master mix (Thermo Fisher, Waltham, MA). All Sanger sequencing was performed by Genewiz, South Plainfield, NJ.

**TABLE 1:** S. aureus bacterial strains used in this study

S. aureus strain and genotype	Description	Reference or source
RN4220		
WT	Restriction enzyme-deficient cloning intermediate strain	43
USA300		
WT	Wild-type, community-acquired methicillin-resistant S. aureus (CA-MRSA) isolate	45
*ΔlcpA*	In-frame, unmarked deletion of *lcpA* (*SAUSA300_1257*) generated by allelic exchange	This work
AAR1		
*lcpA* A757T	Suppressor mutant resistant to arachidonic acid toxicity	This work
JE2		
WT	Wild-type, USA300 community-acquired methicillin-resistant S. aureus (CA-MRSA) isolate	32
*ΔlcpA*	In-frame, unmarked deletion of *lcpA* (*SAUSA300_1257*) generated by allelic exchange	This work
*lcpB*	*lcpB*::*Tn* (*NE1778*; *SAUSA300_0958*::*Tn*)	32
*lcpC*	*lcpC*::*Tn* (*NE415*; *SAUSA300_2259*::*Tn*)	32
*tarO*	Inactive variant of *tarO* lacking 18 bp	30
*tarO ΔlcpA*	Inactive variant of *tarO* lacking 18 bp; in-frame, unmarked deletion of *lcpA* (*SAUSA300_1257*) generated by allelic exchange	This work

**TABLE 2:** Plasmids

Plasmid	Description	Reference or source
pKOR1	Temp-sensitive vector for allelic exchange	42
pKOR1.*ΔlcpA*	Allelic exchange vector for the deletion of *lcpA*	This work
pOS1.P*_lgt_*	Expression vector with *lgt* (constitutive) promoter	44
pOS1.P*_lgt_lcpA*	Expression vector with *lcpA* driven by the *lgt* (constitutive) promoter	This work

**TABLE 3:** Primers

Primer name	Primer sequence
WNB00031	CGTCATACACCAAAAGCAACC
WNB00032	GCTGGCGTAAGAAAGTCAG
WNB00037	ATACGCTTTAGGTGGTCCAG
WNB00041	ATGACCATGTAATACGACTCACTATACATTTCAACGGTCTATATCG
WNB00046	GACGGCCAGTCTTAAGCTCGGGCCCGGGCTGAATTGGCCATAATTTC
WNB00047	ATAGTGAGTCGTATTACATGGTC
WNB00048	GGGCCCGAGCTTAAGACTG
WNB00049	GCCTTGTTTATTTATTTACCTACCTTATATCTTCAAAAATAG
WNB00050	AAGGTAGGTAAATAAATAAACAAGGCGATTTCTATCATAC
WNB00058	CACTAACCTGCCCCGTTAGTTG
WNB00059	ACACTTTATGCTTCCGGCTCG
WNB00066	GACATCCTTTCATTAGACCTT
WNB00067	AACGAACAAGGCGACTTC
WNB00078	CATATGGATAAAGAAACTAATGAC
WNB00079	GGATCCTTAATCTTCATCTAAAA
WNB00080	CCAAAGCGCTAACCTTTTAGC
WNB00081	GTTAGCTCACTCATTAGGCACC
WNB00082	GCACCAAAAGCTGACAAC
WNB00083	GCTTTTCAATGTAGATTGGTG
WNB00084	AATTTCACACAGGAAACAGC
WNB00095	CATCATAGGTATGATTGCATGGT
WNB00096	CTCTGATCTATCCCATTCGC

### S. aureus bacterial cultures.

Unless otherwise noted, S. aureus strains were streaked on TSA and grown 24 h at 37°C. Single bacterial colonies were picked and inoculated into 5 ml TSB in 15-ml polypropylene tubes with aeration lids. Tubes were placed at a 45° angle in an Innova44 shaking incubator (Eppendorf, Hauppauge, NY) set to 37°C with shaking at 180 rpm for 16 h.

### S. aureus kinetic growth curves.

All growth curve experiments were performed on an Epoch 2 plate reader (BioTek, Winooski, VT) at 37°C with linear shaking at 567 cpm (3-mm excursion) for 24 h, taking the optical density at 600 nm (OD_600_) every 30 min, unless otherwise noted. All data were plotted and statistical analyses performed in Prism 7 (GraphPad, La Jolla, CA).

### Allelic exchange.

Allelic exchange was performed using pKOR1 plasmid as described previously (42), with slight modifications with respect to construct design and assembly. The pKOR1 backbone was PCR linearized with primers WNB00047 and WNB00048. The 1-kb upstream flanking region of *lcpA* was PCR amplified using primers WNB00041 and WNB00049, while the 1-kb downstream flanking region was amplified with primers WNB00046 and WNB00050. All primers were designed using the NEBuilder tool and assembled using HiFi master mix as described by the manufacturer (NE BioLabs, Ipswich, MA). The plasmid was chemically transformed into Escherichia coli DH5α and then into the cloning intermediate S. aureus RN4220 (43) before electroporation into the final S. aureus strain. The pKOR1.*ΔlcpA* construct was confirmed by PCR amplification using primers WNB00058 and WNB00059 and Sanger sequencing with primers WNB00066 and WNB00067. Deletions of *lcpA* were confirmed by PCR amplification of genomic DNA using primers WNB00095 and WNB00096 and Sanger sequencing with primers WNB00066 and WNB00067.

### Constitutive expression of *lcpA*.

Plasmid pOS1.P*_lgt_* was linearized by digestion with NdeI and BamHI (NE BioLabs) (44). The lipoprotein diacylglyceryl transferase (*lgt*) promoter was used for constitutive expression of *lcpA*. *lcpA* was amplified by PCR using primers WNB00078 and WNB00079, which added an NdeI digestion site on the 5′ end and a BamHI digestion site on the 3′ end. The construct was assembled using T4 DNA ligase (Promega, Madison, WI) as described by the manufacturer. The plasmid was chemically transformed into E. coli DH5α and then into the cloning intermediate S. aureus RN4220 before electroporation into the final S. aureus strain. The pOS1.P*_lgt_lcpA* construct was confirmed by PCR amplification using primers WNB00080 and WNB00081 and Sanger sequencing with primers WNB00082, WNB00083, and WNB00084.

### AA concentration-dependent growth inhibition of S. aureus.

USA300 (45) was diluted 1,000-fold into 100 μl TSB containing 0, 50, 100, or 200 μM AA, and growth was monitored in biological triplicate as described above.

### Time course of AA treatment CFU enumeration.

USA300 was diluted 1,000-fold into 200 μl TSB containing 0 or 400 μM AA in biological triplicate. The plate was incubated in an Innova44 incubator by shaking as described above. At 0, 2, 4, 6, and 8 h postinoculation, 10 μl was removed from each sample and serially diluted in 10-fold intervals in phosphate-buffered saline (PBS). Each dilution was spot plated on TSA and incubated overnight at 37°C followed by CFU enumeration.

### Paraquat, Mn, and AA growth curve.

USA300 was diluted 1,000-fold into 100 μl TSB containing 0 or 100 μM AA, 0 or 250 μM MnCl_2_, and 0 or 250 μM paraquat, and growth was monitored in biological triplicate as described above.

### AA growth curve with and without α-tocopherol.

USA300 was diluted 1,000-fold into 100 μl TSB containing 0, 100, or 200 μM AA and 0 or 80 μM α-tocopherol, and growth was monitored in biological triplicate as described above.

### Pyridoxamine, 5′-*O*-pentyl-pyridoxamine, and AA growth curve.

USA300 was diluted 1,000-fold into 100 μl TSB containing 0 or 200 μM AA, 0 or 1 mM PM, and 0 or 1 mM PnPM, and growth was monitored in biological triplicate as described above.

### Isolevuglandin modulation by PnPM Western blotting.

USA300 was diluted 20-fold into 5 ml TSB containing 0 or 400 μM AA and 0 or 5 mM PnPM. Samples were incubated with shaking for 4 h in an Innova44 incubator as described above. After incubation, cells were pelleted in a table top centrifuge. Cell pellets were suspended in 500 μl PBS containing 10 mM MgCl_2_ and 20 μg lysostaphin (Ambi, Lawrence, NY) and incubated 30 min at 37°C. IGEPAL was added to each sample to reach a final concentration of 2% (vol/vol) and incubated 15 min on ice. Each sample was sonicated with a model 150E Ultrasonic Dismembrator (Fisher Scientific, Waltham, MA) three times, with resting on ice for 10 min between sonications. Insoluble debris was pelleted in a microcentrifuge (Eppendorf) at maximum speed for 10 min at 4°C. Protein in the lysate was quantified by bicinchoninic acid (BCA) assay (Thermo Fisher). Samples were normalized to protein concentrations, and 10.5 μg of each sample was separated on a 4% to 20% polyacrylamide gel (Bio-Rad, Hercules, CA). The gel was transferred to 0.45-μm-pore-size nitrocellulose in a Trans-Blot Turbo system (Bio-Rad) and maintained for 17 min at 25 V. The blot was stained with Ponceau S to ensure consistent loading and was then blocked with protein-free (PBS) blocking buffer (Thermo Fisher). D11 ScFv antibody, which binds to IsoLG PTM (27), was diluted 500-fold in protein-free (PBS) blocking buffer and rocked with the blot overnight at 4°C. After washing was performed, anti-E tag (rabbit) antibody (no. 1; Abcam, Cambridge, United Kingdom) was diluted 1,000-fold in protein-free (PBS) blocking buffer and rocked with the blot overnight at 4°C. After washing, anti-rabbit IRDye 800CW Ab (no. 2; Li-Cor, Lincoln, NE) was diluted 5,000-fold in protein-free (PBS) blocking buffer and rocked for 1 h at 25°C. After washing was performed, the blot was scanned on an Odyssey Imager (Li-Cor).

### CFU enumeration with PnPM and AA.

USA300 was diluted 1,000-fold into 200 μl TSB containing 0 or 1 mM PnPM and 0 or 100 μM AA in biological triplicate. The plate was incubated with shaking in an Innova44 incubator as described above. At 0 and 6 h postinoculation, 10 μl was removed from each sample and serially diluted in 10-fold intervals in PBS. Each dilution was spot plated on TSA and incubated overnight at 37°C followed by CFU enumeration.

### Selection of AA-resistant mutants.

USA300 was diluted 5,000-fold into TSB plus 200 μM AA and incubated with shaking for 4 h at 37°C. The sample was streaked on TSA plus 200 μM AA and incubated overnight at 37°C. Colonies were picked and streaked on TSA plus 200 μM AA and incubated overnight at 37°C five more times. After the selection, colonies were picked and streaked on TSA and incubated overnight at 37°C, and the procedure was repeated five more times. The resulting strains were subjected to kinetic growth curve analysis as described above to determine which strains contained stable mutations that conferred resistance to AA stress.

### Whole-genome sequencing of AA-resistant mutant.

USA300 and AAR1 genomic DNA was isolated using a Wizard genomic DNA kit (Promega) and quantified using a Quant-It kit (Invitrogen). Library preparation and sequencing were performed at Vanderbilt Technologies for Advanced Genomics (VANTAGE). The library was prepared using a TrueSeq DNA Nano kit (Illumina, San Diego, CA), and the sequencing was performed with 75-bp paired ends on an Illumina Hi-Seq 3000 sequencer. Mutations compared to the S. aureus TCH1516 genome were called using CLC Genomics Workbench 9.0 (CLC bio, Aarhus, Denmark) at the Tufts University Genomics Core. The mutation discovered in *lcpA* was validated by generating a PCR product of the gene using WNB00031 and WNB00032 and by Sanger sequencing using WNB00037. Alignment of the wild-type (WT) and AA-resistant mutant LcpA proteins was performed in CLC Sequence Viewer 8 (CLC bio).

### D11 Western blotting of WT and AAR1 S. aureus with and without AA treatment.

USA300 and AAR1 were diluted 20-fold into 5 ml TSB containing 0 or 400 μM AA. Samples were incubated with shaking for 4 h in an Innova44 incubator as described above. After incubation, cells were pelleted in a table top centrifuge. Cell pellets were suspended in 500 μl PBS containing 10 mM MgCl_2_ and 20 μg lysostaphin and incubated 30 min at 37°C. IGEPAL was added to each sample to reach a final concentration of 2% (vol/vol) and incubated 15 min on ice. Each sample was sonicated with a model 150E Ultrasonic Dismembrator three times, with resting on ice for 10 min between sonications. Insoluble debris was pelleted in a microcentrifuge at maximum speed and 4°C for 10 min. Protein in the lysate was quantified by BCA assay. Samples were normalized to protein concentration, and 10.5 μg of each sample was separated on a 4% to 20% gradient polyacrylamide gel. The gel was transferred to 0.45-μm-pore-size nitrocellulose in a Trans-Blot Turbo system at 25 V for 17 min. The blot was stained with Ponceau S to ensure consistent loading and was then blocked with protein-free (PBS) blocking buffer. D11 ScFv antibody (27) was diluted 500-fold in protein-free (PBS) blocking buffer and rocked with the blot overnight at 4°C. After washing was performed, anti-Etag (rabbit) antibody no. 1 was diluted 1,000-fold in protein-free (PBS) blocking buffer and rocked with the blot overnight at 4°C. After washing was performed, anti-rabbit IRDye 800CW Ab no. 2 was diluted 5,000-fold in protein-free (PBS) blocking buffer and rocked for 1 h at 25°C. After washing was performed, the blot was scanned on an Odyssey Imager.

### AA growth curve with *lcpA* overexpression.

Strains USA300 pOS1.P*_lgt_*, AAR1 pOS1.P*_lgt_*, USA300 *ΔlcpA* pOS1.P*_lgt_*, USA300 pOS1.P*_lgt_lcpA*, AAR1 pOS1.P*_lgt_lcpA*, and USA300 *ΔlcpA* pOS1.P*_lgt_lcpA* were streaked on TSA plus 10 μg/ml chloramphenicol and grown 24 h at 37°C. Individual colonies were inoculated into TSB plus 10 μg/ml chloramphenicol in biological triplicate and incubated with shaking for 16 h at 37°C. Each bacterial strain was diluted 1,000-fold into 100 μl TSB containing 10 μg/ml chloramphenicol and 0 or 100 μM AA, and growth was monitored as described above.

### CFU enumeration in USA300 and the USA300 *ΔlcpA* mutant treated with AA.

USA300 and the USA300 *ΔlcpA* mutant were diluted 1,000-fold into 100 μl TSB containing 0 or 100 μM AA in biological triplicate. The plate was incubated in an Innova44 incubator with shaking for 6 h as described above. Each sample was serially diluted in 10-fold intervals in PBS. Each dilution was spot plated on TSA and incubated overnight at 37°C followed by CFU enumeration.

### AA growth curve in strains lacking individual *lcp* genes.

Strains JE2, which is USA300 cured for erythromycin resistance (32), JE2 *ΔlcpA*, JE2 *lcpB* (32), and JE2 *lcpC* (32) were diluted 1,000-fold into 100 μl TSB containing 0 or 50 μM AA, and growth was monitored in biological triplicate as described above.

### Beta-lactam sensitivity comparing MSSA and MRSA strains with and without *lcpA* inactivated.

Strain USA300, the USA300 *ΔlcpA* mutant, and strain Newman were diluted 1,000-fold into 100 μl TSB containing 0 or 2 μg/ml oxacillin, and growth was monitored in biological quadruplicate as described above.

### Beta-lactam sensitivity with *lcpA* constitutive expression.

Strains USA300 pOS1.P*_lgt_*, USA300 *ΔlcpA* pOS1.P*_lgt_*, USA300 pOS1.P*_lgt_lcpA*, and USA300 *ΔlcpA* pOS1.P*_lgt_lcpA* were streaked on TSA plus 10 μg/ml chloramphenicol and grown 24 h at 37°C. Individual colonies were inoculated into TSB plus 10 μg/ml chloramphenicol in biological triplicate and incubated with shaking for 16 h at 37°C. Each bacterial strain was diluted 1,000-fold into 100 μl TSB containing 10 μM chloramphenicol and 0 or 4 μg/ml oxacillin, and growth was monitored as described above.

### Membrane permeability of USA300 and the USA300 *ΔlcpA* mutant with AA.

USA300 and the USA300 *ΔlcpA* mutant were diluted 10-fold into 100 μl TSB containing 0 or 200 μM AA and 0 or 100 μM diphenylhexatriene in biological quadruplicate. Plate growth was maintained at 37°C on a Cytation 5 system with linear shaking at 567 cpm (3 mm). Optical density at 600 nm and fluorescence (excitation, 365 nm; emission, 460 nm) were measured at 20-min intervals. The data displayed were background corrected for the wells with all components except cells and normalized to OD_600_.

### AA incorporation into USA300 and the USA300 *ΔlcpA* mutant.

USA300 and the USA300 *ΔlcpA* mutant were diluted 5-fold in 5 ml TSB containing 0 or 400 μM AA and incubated with shaking for 1 h in biological quadruplicate. Each sample was split into two 500-μl aliquots, and the cells were pelleted and washed with 1 ml PBS. All pellets were suspended in 200 μl PBS containing 10 mM MgCl_2_ and 10 μg lysostaphin and incubated 30 min at 37°C.

### Extraction of free AA from S. aureus.

To one set of samples, 1 ml ethyl acetate containing 10 nmol AA-*d_11_* was added to extract the free AA (46). The ethyl acetate layer was removed and dried under a stream of N_2_ at 37°C. Each sample was suspended in 300 μl 1:1 methanol/0.1% formic acid for liquid chromatography/tandem mass spectrometry (LC-MS/MS) analysis.

### Extraction of free and phospholipid-esterified AA from S. aureus.

To the other set of samples, 1 ml 4:6 methanol/chloroform containing 10 nmol AA-*d_11_* was added to extract both the free and phospholipid-esterified AA (47). The chloroform layer was removed and dried under a stream of N_2_ at 37°C. Each sample was suspended in 500 μl methanol and then added to 2 ml 1 N NaOH and incubated 2 h at 37°C to hydrolyze esterified AA (47). To each sample, 5 ml of ethyl acetate was added to extract the free AA. The ethyl acetate layer was removed and dried under a stream of N_2_ at 37°C. Each sample was suspended in 300 μl 1:1 methanol/0.1% formic acid for LC-MS/MS analysis.

### LC-MS/MS quantification of AA levels.

AA and AA-*d_11_* were analyzed on a Thermo TSQ Quantum Ultra system with an electrospray ionization (ESI) source with a skimmer offset of 24 V, determined empirically, and interfaced to a Waters Acquity ultraperformance liquid chromatography (UPLC) system. Analytes were separated using a gradient and high-performance liquid chromatography (HPLC) on a Supelco Ascentis Express C_18_ column (50 by 2.1 mm, 5-μm pore size) at a flow rate of 0.5 ml/min using 0.1% formic acid–water and 0.1% formic acid–acetonitrile as the A and B mobile phases, respectively. The gradient was held at 50% mobile phase B for 1 min and then ramped to 100% mobile phase B over the next 2 min. The column was washed at 100% mobile phase B for 3 min and equilibrated to 50% mobile phase B for 3 min. The analytes were measured by selected-reaction monitoring (SRM) in negative-ionization mode at 303.2 to 259.2 *m/z* transition for AA and 314.2 to 270.2 *m/z* transition for AA-*d_11_* using a collision energy setting of 14 V and a scan time of 100 ms. AA was quantified by comparing the area under the concentration-time curve (AUC) determined for AA to the AUC determined for AA-*d_11_*.

### AA growth curve with tunicamycin treatment.

USA300 and the USA300 *ΔlcpA* mutant were diluted 1,000-fold into 100 μl TSB containing 0 or 50 μM AA and 0 or 2 μg/ml tunicamycin (Cayman Chemical), and growth was monitored in biological triplicate as described above.

### AA growth curve in wall teichoic acid biosynthesis mutants.

JE2 and the JE2 *ΔlcpA* mutant were diluted 1,000-fold and JE2 *tarO* (30) and the JE2 *tarO ΔlcpA* mutant were diluted 100-fold into 100 μl TSB containing 0 or 100 μM AA, and growth was monitored in biological triplicate as described above. The dilution differences between the strains were used because strains lacking *tarO* do not grow to the same stationary-phase confluence as the other strains (See Fig. 6C for CFU data).

### D11 Western blotting of JE2, JE2 *ΔlcpA*, and JE2 *tarO* S. aureus with and without AA treatment.

JE2 and the JE2 *ΔlcpA* mutant were diluted 20-fold into 5 ml TSB containing 0 or 400 μM AA, and JE2 *tarO* was diluted 2-fold into 5 ml TSB containing 0 or 400 μM AA. Samples were incubated with shaking for 4 h in an Innova44 incubator as described above. After incubation, cells were pelleted in a table top centrifuge. Cell pellets were suspended in 500 μl PBS containing 10 mM MgCl_2_ and 20 μg lysostaphin and incubated 30 min at 37°C. IGEPAL was added to each sample to reach a final concentration of 2% (vol/vol) and incubated 15 min on ice. Each sample was sonicated with a model 150E Ultrasonic Dismembrator three times, with resting on ice for 10 min between sonications. Insoluble debris was pelleted in a microcentrifuge at maximum speed and 4°C for 10 min. Protein in the lysate was quantified by BCA assay. Samples were normalized to protein concentration, and 10.5 μg of each sample was separated on a 4% to 20% gradient polyacrylamide gel. The gel was transferred to 0.45-μm-pore-size nitrocellulose in a Trans-Blot Turbo system at 25 V for 17 min. The blot was stained with Ponceau S to ensure consistent loading and was then blocked with protein-free (PBS) blocking buffer. D11 ScFv antibody (27) was diluted 500-fold in protein-free (PBS) blocking buffer and rocked with the blot overnight at 4°C. After washing was performed, anti-Etag (rabbit) antibody no. 1 was diluted 1,000-fold in protein-free (PBS) blocking buffer and rocked with the blot overnight at 4°C. After washing was performed, anti-rabbit IRDye 800CW Ab no. 2 was diluted 5,000-fold in protein-free (PBS) blocking buffer and rocked for 1 h at 25°C. After washing was performed, the blot was scanned on an Odyssey Imager.

### AA growth curve of JE2 *tarO* with and without α-tocopherol.

JE2 *tarO* was diluted 100-fold into 100 μl TSB containing 0 μM or 25 μM AA and 0 μM or 80 μM α-tocopherol, and growth was monitored in biological triplicate as described above.

### CFU enumeration in wall teichoic acid mutants with and without AA.

JE2 and the JE2 *ΔlcpA* mutant were diluted 1,000-fold and JE2 *tarO* and the JE2 *tarO ΔlcpA* mutant were diluted 100-fold into 100 μl TSB containing 0, 25, 50, or 100 μM AA in biological triplicate. The plate was incubated in an Innova44 incubator with shaking for 3 h as described above. Each sample was serially diluted in 10-fold intervals in PBS. Each dilution was spot plated on TSA and incubated overnight at 37°C followed by CFU enumeration.

### Reactive oxygen species measurements with and without AA.

Strains JE2, JE2 *ΔlcpA*, and JE2 *tarO* were diluted 10-fold into 100 μl TSB containing 0 or 100 μM AA and 0 or 50 μM dihydrorhodamine 123 (Invitrogen, Carlsbad, CA) in biological quadruplicate. Plate growth was maintained at 37°C on a Cytation 5 system (BioTek) with linear shaking at 567 cpm (3 mm). Optical density at 600 nm and fluorescence (excitation, 510 nm; emission, 580 nm) were measured at 10-min intervals. The data displayed were background corrected for the wells with all components except cells and normalized to OD_600_.

### Neutrophil killing.

USA300 and the USA300 *ΔlcpA* mutant were diluted 100-fold in non-heat-inactivated fetal bovine serum (FBS) and placed on ice for 1 h to allow opsonization in biological triplicate. Bone marrow was isolated from the femurs and tibias of four 7-week-old female BALB/cJ mice (The Jackson Laboratory, Bar Harbor, ME). Neutrophils were isolated from the bone marrow using density centrifugation (48), and 10^4^ neutrophils were transferred to a low-attachment, round-bottom 96-well plate to rest for 1 h at 37°C and 5% CO_2_ in 250 μl D10 media (Dulbecco’s modified Eagle’s medium [DMEM] plus 10% FBS). Opsonized bacteria were transferred to the plates containing neutrophils (multiplicity of infection [MOI] of 2) or lacking neutrophils and incubated at 37°C and 5% CO_2_. At 0.5, 2, 6, 12, and 24 h, the wells containing bacteria were serially diluted and the dilutions were spot plated to TSA and incubated overnight at 37°C followed by CFU enumeration. Percent growth was quantified by comparing the CFUs enumerated from the plates containing neutrophils to the CFUs enumerated from the plates without neutrophils.

### Quantification of AA released in S. aureus activated neutrophils.

Bone marrow was isolated from the femurs and tibias of four 8-week-old female BALB/cJ mice (The Jackson Laboratory, Bar Harbor, ME). Neutrophils were isolated from the bone marrow using density centrifugation (48), and 2 × 10^6^ neutrophils were transferred to a low-attachment, round-bottom 96-well plate to rest for 1.5 h at 37°C and 5% CO_2_ in 250 μl D10 media (DMEM plus 10% FBS). USA300 was diluted to 2 × 10^6^ bacteria per 5 μl in PBS. Bacteria were heat killed by incubation for 20 min at 90°C. Heat-killed bacteria were transferred to the plates containing neutrophils (MOI of 1) and incubated at 37°C and 5% CO_2_. At 2, 4, and 6 h after bacterial dilution, the entire content of each well was transferred to 5 ml ethyl acetate containing 500 pmol AA-*d_11_*. The ethyl acetate layer was removed and dried under a stream of N_2_ at 37°C. Each sample was suspended in 200 μl 1:1 methanol/0.1% formic acid for LC-MS/MS analysis as described above.

### Strain USA300 and USA300 *ΔlcpA* killing by HOCl.

USA300 and the USA300 *ΔlcpA* mutant were diluted 100-fold into 100 μl PBS containing 0, 12.5, 25, or 50 μM NaOCl (Clorox, Oakland, CA) in biological triplicate. The plate was incubated at 25°C for 30 min. Each sample was serially diluted in 10-fold intervals in PBS. Each dilution was spot plated on TSA and incubated overnight at 37°C followed by CFU enumeration.

### Strain USA300 and USA300 *ΔlcpA* growth inhibition by calprotectin.

Recombinant calprotectin was produced as described elsewhere (49). USA300 and the USA300 *ΔlcpA* mutant were diluted 1,000-fold into 100 μl TSB plus 1 mM CaCl_2_ plus 1 mM β-mercaptoethanol (βME) containing 0 or 800 μg/ml calprotectin, and growth was monitored in biological quadruplicate as described above.

### Neutrophil activation by USA300 and the USA300 *ΔlcpA* mutant.

USA300 and the USA300 *ΔlcpA* mutant or sterile filtered supernatant from overnight cultures was diluted 10-fold in non-heat-inactivated FBS and placed on ice for 1 h to allow opsonization. Bone marrow was isolated from the femurs and tibias of two 7-week-old female BALB/cJ mice. Neutrophils were isolated from the bone marrow using density centrifugation (48), and 10^4^ neutrophils were transferred to a low-attachment, round-bottom 96-well plate to rest for 1 h at 37°C and 5% CO_2_ in 250 μl D10 media. Opsonized bacteria were transferred to the plates containing neutrophils (MOI of 10) and incubated at 37°C and 5% CO_2_. At 20 min prior to fixation, dihydrorhodamine 123 and a Live/Dead stain (catalog no. L23105; Invitrogen) were added to the neutrophils. After coculturing of the neutrophils with the bacteria for 30 or 60 min, neutrophils were fixed in 4% paraformaldehyde (PFA) at room temperature and transferred onto ice for 15 min. Neutrophils were spun and aspirated, and cells were resuspended in mouse Fc Block (Tonbo Biosciences; clone 2.4G2) diluted in fluorescence-activated cell sorter (FACS) media (PBS plus 2% FBS plus 0.02% sodium azide) for 20 min on ice. Neutrophils were spun, aspirated, and resuspended in FACS media containing anti-Ly6G (BioLegend) and anti-CD11b (BioLegend) antibodies for 20 min on ice. Finally, neutrophils were spun, aspirated, and resuspended in FACS media for flow cytometry. Data were collected using a BD LSRII flow cytometer with FACSDIVA software and analyzed using FlowJo.

### Murine systemic infection with USA300 and the USA300 *ΔlcpA* mutant.

Mouse experiments were approved by the Vanderbilt University Institutional Animal Care and Use Committee, and experiments were performed according to institutional policies, the Animal Welfare Act, National Institutes of Health guidelines, and American Veterinary Medical Association guidelines on euthanasia. USA300 and the USA300 *ΔlcpA* mutant were diluted 100-fold into TSB and grown for 3 h at 37°C on a roller drum at setting 6 to reach the mid-exponential phase. Cells were pelleted at 5,000 rpm and 4°C for 10 min in a centrifuge (Sorvall, Waltham, MA) and washed with 10 ml ice-cold PBS. The optical density at 600 nm of each culture was adjusted to 0.4 with ice-cold PBS. Seven-week-old female BALB/cJ mice were anesthetized intraperitoneally with 2,2,2-tribromoethanol diluted in PBS. Following anesthetization, the mice were injected retro-orbitally with 4 × 10^7^ CFU of each culture in 100 μl PBS. Mice were monitored twice per day for 4 days. On the fourth day, the mice were euthanized by CO_2_ asphyxiation, and the brain, heart, kidneys, liver, lungs, and spleen were removed. The livers were added individually to Whirl-Pak bags (Nasco, Fort Atkinson, WI) containing 1 ml PBS and homogenized with 100 strokes of a rolling pin at 4°C. Brain, heart, kidneys, and spleen were added individually to 1.5-ml Navy Bullet Blender tubes containing 550 μl PBS and homogenized on a Bullet Blender (Next Advance, Troy, NY) at 4°C on setting 8 for 5 min and then on setting 12 for 5 min. Lungs were added individually to 1.5-ml Navy Bullet Blender tubes containing 800 μl PBS and homogenized on a Bullet Blender at 4°C on setting 8 for 5 min and then on setting 12 for 5 min. Each sample was serially diluted at 10-fold intervals in PBS. Each dilution was spot plated on TSA and incubated overnight at 37°C followed by CFU enumeration.
