# Supplementary figures and images for: Arachidonic Acid Kills Staphylococcus aureus through a Lipid Peroxidation Mechanism
Source: mBio. 2019 Oct 1;10(5):e01333-19. doi: 10.1128/mBio.01333-19 (PMC6775451; doi:10.1128/mBio.01333-19)

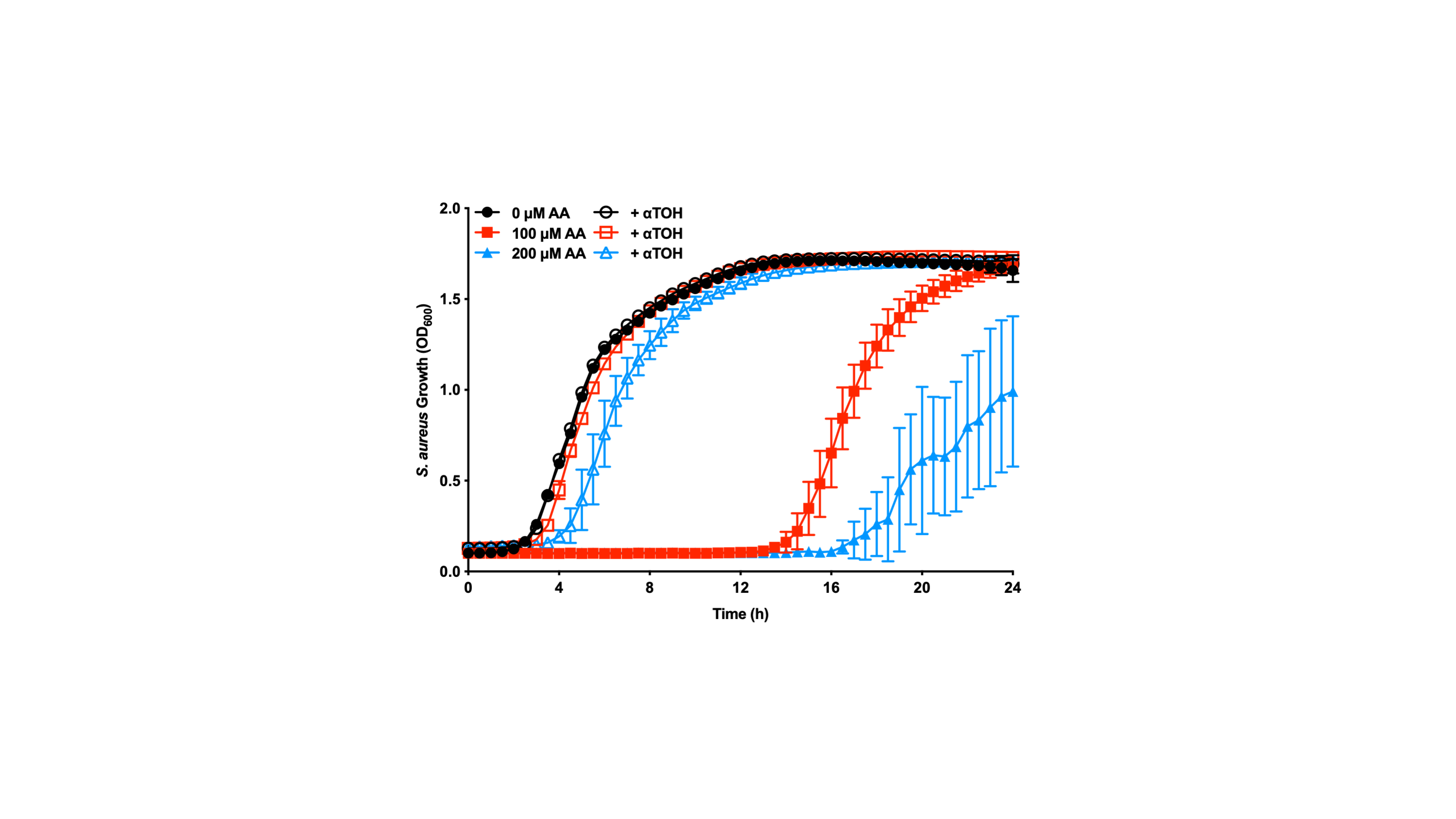

Supplement: FIG S1 [file mBio.01333-19-sf001.tif]

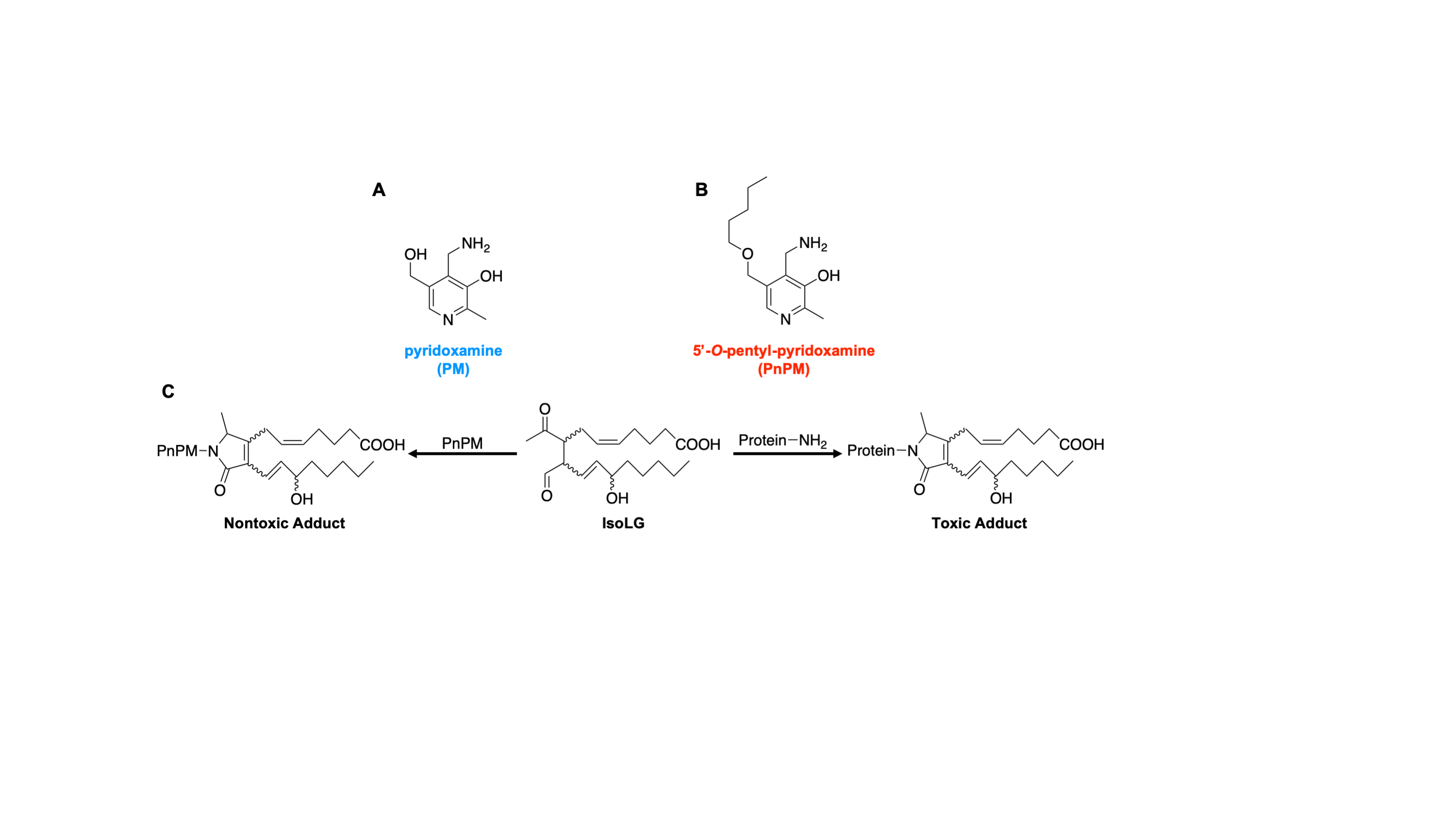

Supplement: FIG S2 [file mBio.01333-19-sf002.tif]

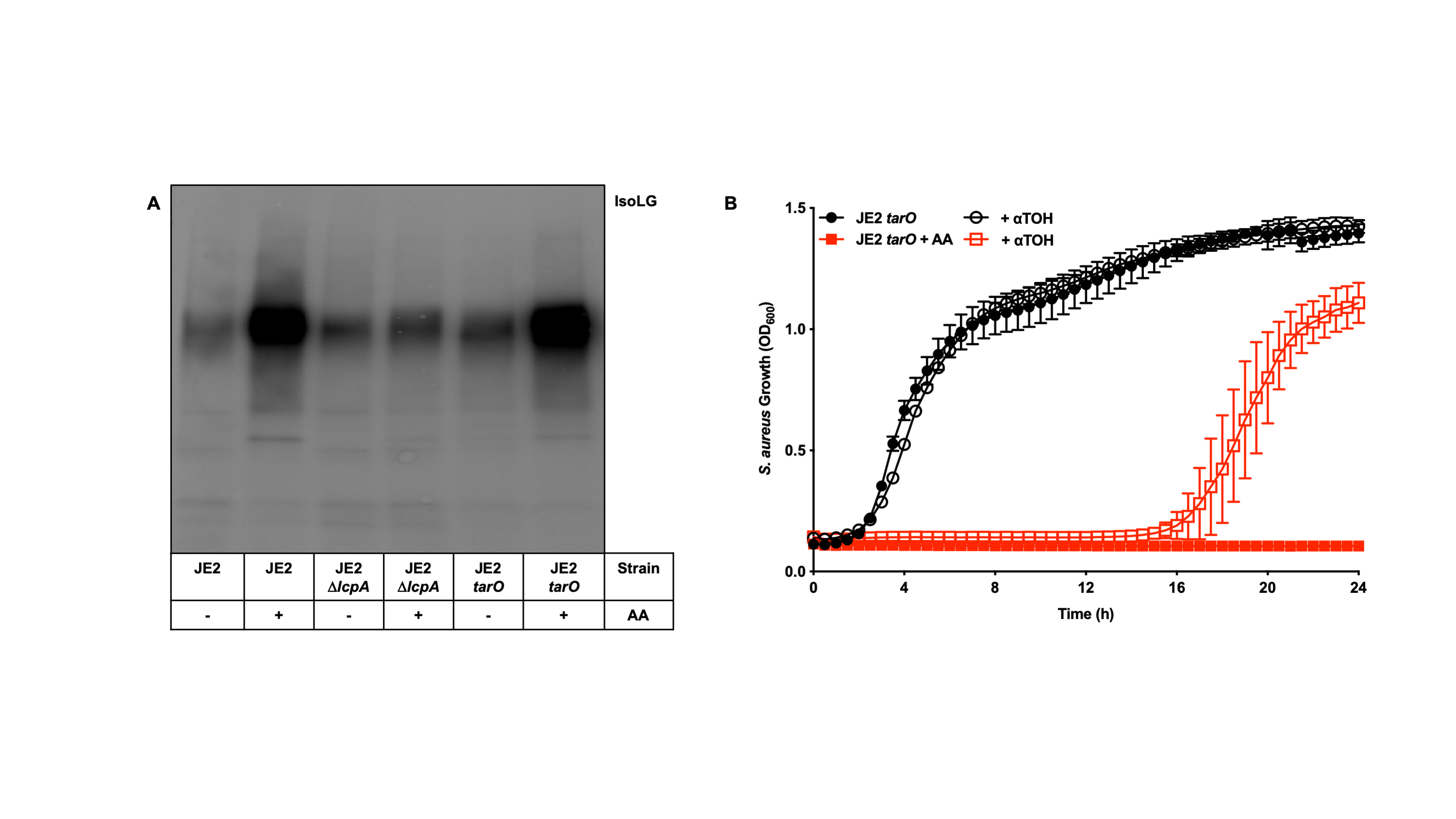

Supplement: FIG S3 [file mBio.01333-19-sf003.tif]

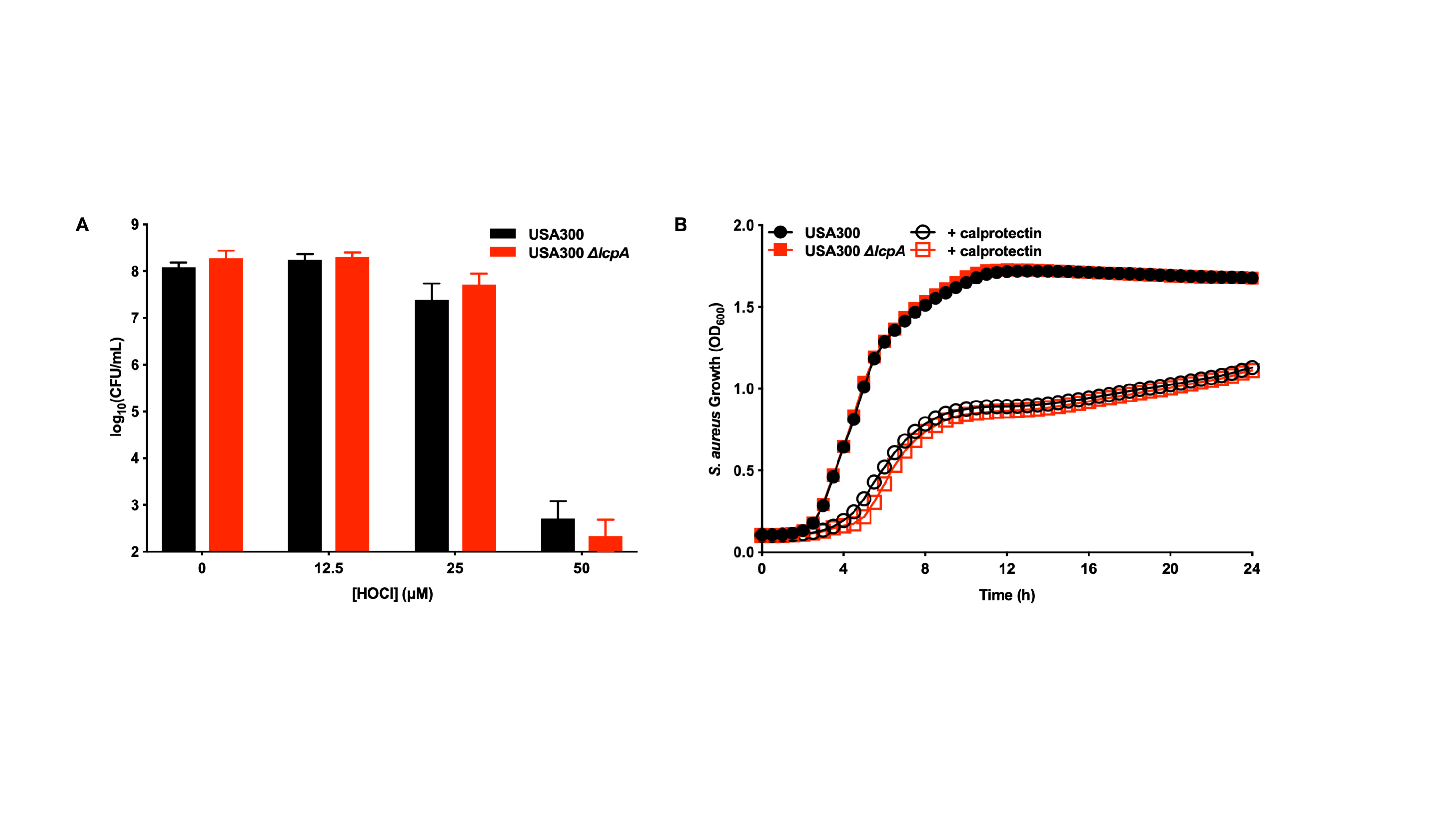

Supplement: FIG S4 [file mBio.01333-19-sf004.tif]

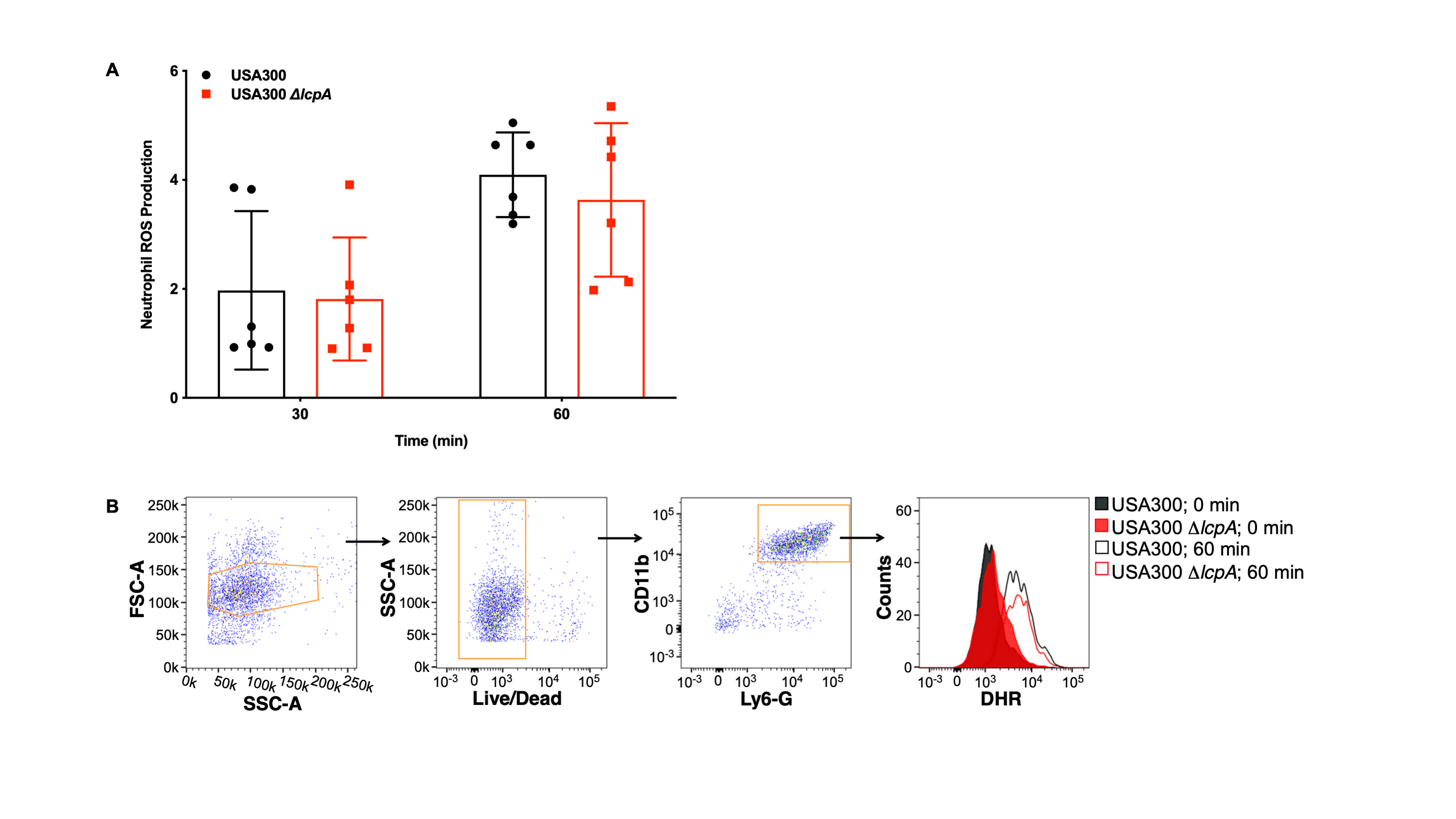

Supplement: FIG S5 [file mBio.01333-19-sf005.tif]

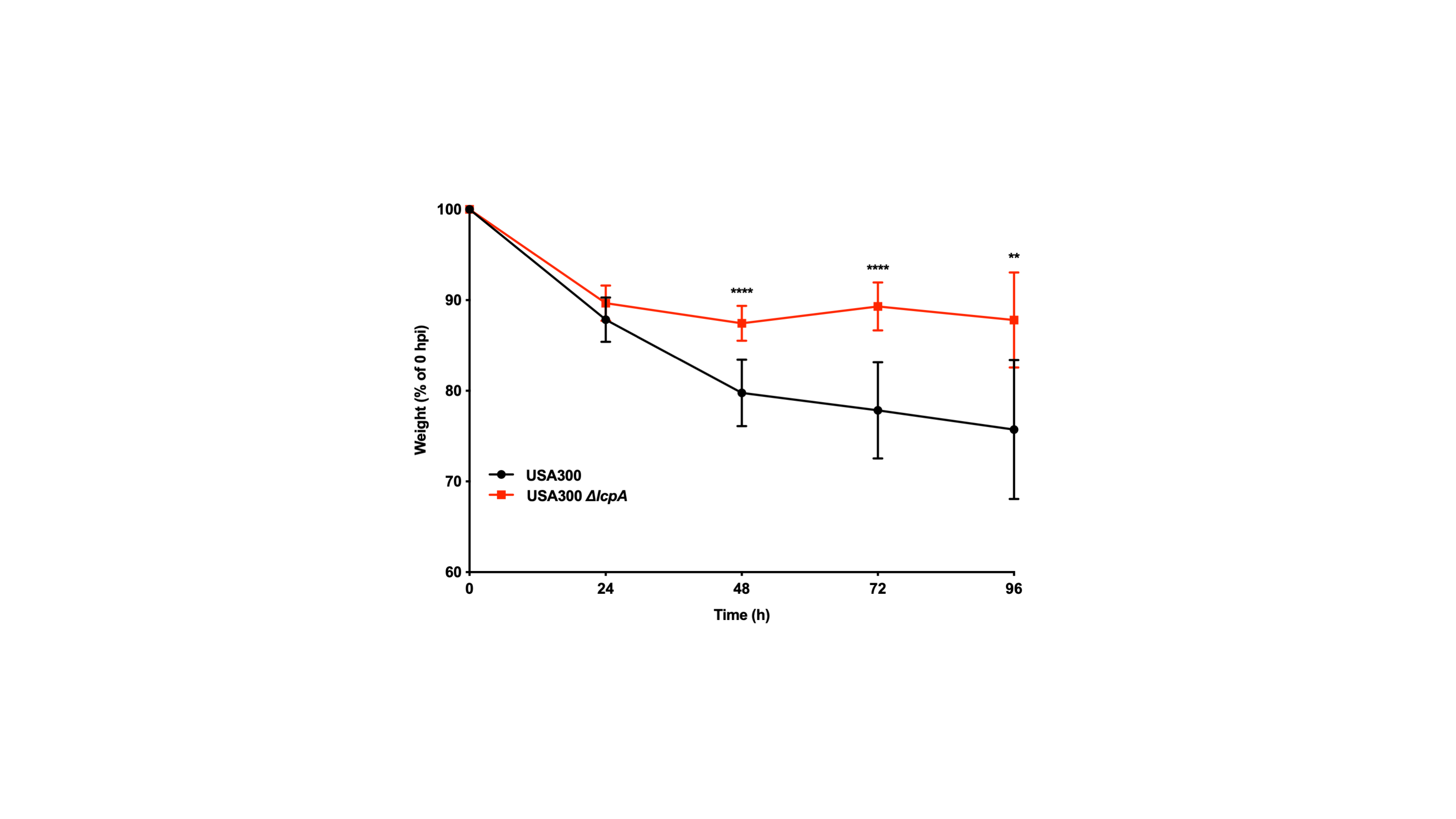

Supplement: FIG S6 [file mBio.01333-19-sf006.tif]
